# Prevalence and correlates of depression and quality of life among primary caregivers of patients with schizophrenia attending a Nigerian Tertiary Hospital

**DOI:** 10.1192/bjo.2021.72

**Published:** 2021-06-18

**Authors:** Akinloye Akinfala, Oladipo Sowunmi, Imam Sakeeb

**Affiliations:** 1 Lancashire & South Cumbria NHS Foundation Trust; 2 Neuropsychiatric Hospital Aro

## Abstract

**Aims:**

To determine the prevalence and correlates of depression and quality of life and their relationship among primary caregivers of patients with schizophrenia in a psychiatry specialist hospital.

**Method:**

A total of 138 caregivers of patients diagnosed with schizophrenia attending the outpatient clinic of the Neuropsychiatric Hospital Aro, Abeokuta were recruited. Sociodemographic questionnaire, Mini International Neuropsychiatric Interview (MINI-PLUS) (depressive module) and World Health Organization Quality of Life-Bref (WHOQOL-Bref) were administered on the caregivers while Brief Psychiatric Rating Scale (BPRS) was used to measure symptoms severity in the patients.

**Result:**

The mean (±SD) age of respondents was 48.3 years (±14.7), 53.6% were females and 33.3% were without partners. The prevalence of depression among the caregivers who participated in the study was 13.8%. Female gender (χ2 = 5.68, df = 1, p = 0.02), hailing from a minority tribe (χ2 = 9.78 df = 1, p < 0.01), and Previous treatment for mental illness (χ2 = 8.24 df = 1, p < 0.01) were associated with depression. Female gender (ß = 1.35, OR = 3.86, p = 0.03), minority tribe (ß = 1.95, OR = 7.03, p < 0.01), and previous treatment for mental illness (ß = 3.19, OR = 24.21, p = 0.01) were independently predictive of depression in the caregivers.

Independent predictors of lower quality of life (QOL) were: Parents/siblings relationship for social relationship domain (ß = −7.076, p = 0.037) and spending more than 35 hours per week for Environmental domain (ß = −5.622, p = 0.028).

Finally, a significant correlation was also found between Depression and Psychological Domain of QOL (t = 3.048, p < 0.01) and Social Domain of QOL (t = 2.154, p = 0.03).

**Conclusion:**

This study shows that primary caregivers of patients with schizophrenia have high prevalence of depression and poor quality of life. There is need to pay attention to the psychological wellbeing and quality of life of caregivers who come in contact with psychiatric services, and not just the patients they accompany.

